# 754 Venous Thromboembolism Incidence and Risk Factors in Burn Patients

**DOI:** 10.1093/jbcr/irae036.296

**Published:** 2024-04-17

**Authors:** Eloise Stanton, Haig A Yenikomshian, Justin Gillenwater

**Affiliations:** Keck School of Medicine of USC, Los Angeles, CA; University of Southern California, Los Angeles, CA; Keck Medicine of USC, Los Angeles, CA; Keck School of Medicine of USC, Los Angeles, CA; University of Southern California, Los Angeles, CA; Keck Medicine of USC, Los Angeles, CA; Keck School of Medicine of USC, Los Angeles, CA; University of Southern California, Los Angeles, CA; Keck Medicine of USC, Los Angeles, CA

## Abstract

**Introduction:**

In burn care, there is a noticeable lack of comprehensive studies on the incidence and risk factors related to Venous Thromboembolism (VTE) in burn patients. Understanding VTE in this context is crucial, as it can lead to severe complications and patient morbidity/mortality. This abstract investigates VTE incidence and risk factors in burn patients in hopes of informing VTE prevention measures and improving overall patient care in the field of burn medicine.

**Methods:**

We conducted an analysis of the US National Trauma Data Bank (NTDB) spanning from 2007 to 2021 to identify burn-injured patients. Our primary focus was on determining the incidence of VTE. As predictor variables, we considered factors such as age, sex, smoking status, history of anticoagulation use, diabetes, and prior history of stroke. We abstracted comorbidities using event codes within the database.

**Results:**

442,623 patients were identified who had sustained burn injuries within the NTDB between 2007 to 2021. Among this cohort, 7,384 individuals (approximately 1.7%) experienced a VTE event during their hospitalization. Of these, 4,138 (56.0%) were deep vein thromboses and 1,504 (20.4%) were pulmonary emboli. Patients with VTE were significantly older than those without (37.7 versus 35.2, p < .001) and were significantly more likely to be male (82.5% vs. 78.9% p < .001) and obese (7.4% vs. 3.4% p < .001). Patients with VTE also had significantly higher %TBSA burns (16.5% vs. 13.2%, p < .001). Patients with VTE were also significantly more likely to be smokers (28.9% vs. 26.5%, p=.025), have preexisting hypertension (9.0% vs. 8.1%, p=.002), have a previous myocardial infarction (7.5% vs. 4.8%, p < .001), have alcohol use disorder (14.0% vs. 6.6%, p < .001) or have substance abuse (31.7% vs. 20.1%, p < .001). There were no signficant differences between the two cohorts in regard to history of stroke, peripheral vascular disease, diabetes mellitus, steroid use, or renal disease (Table 1).

**Conclusions:**

This study sheds light on several risk factors, including age, obesity, male sex, and comorbidities such as hypertension, myocardial infarction, and substance use disorders, which merit attention in clinical practice when selecting VTE prevention strategies. Addressing these factors and developing tailored interventions can help improve patient care, mitigate complications, and enhance outcomes. This study underscores the importance of considering VTE risk in burn patients as an integral component of comprehensive burn care.

**Applicability of Research to Practice:**

By identifying specific risk factors and highlighting the incidence of VTE among burn patients, healthcare providers can implement more targeted VTE prevention and management strategies. This knowledge empowers clinicians to make informed decisions, ensuring that patients receive timely and tailored interventions, ultimately enhancing the quality of care and patient outcomes in the management of burn injuries.

